# Cocoa Diet and Antibody Immune Response in Preclinical Studies

**DOI:** 10.3389/fnut.2017.00028

**Published:** 2017-06-27

**Authors:** Mariona Camps-Bossacoma, Malen Massot-Cladera, Mar Abril-Gil, Angels Franch, Francisco J. Pérez-Cano, Margarida Castell

**Affiliations:** ^1^Faculty of Pharmacy and Food Science, Department of Biochemistry and Physiology, Section of Physiology, University of Barcelona, Barcelona, Spain; ^2^Nutrition and Food Safety Research Institute (INSA-UB), Santa Coloma de Gramenet, Spain

**Keywords:** gut-associated lymphoid tissue, IgA, IgG, IgM, immunoregulator, lymph nodes, spleen, tolerance

## Abstract

The ability of cocoa to interact with the immune system *in vitro* and *in vivo* has been described. In the latter context, a cocoa-enriched diet in healthy rats was able to modify the immune system’s functionality. This fact could be observed in the composition and functionality of lymphoid tissues, such as the thymus, spleen, and lymph nodes. Consequently, immune effector mechanisms, such as antibody synthesis, were modified. A cocoa-enriched diet in young rats was able to attenuate the serum levels of immunoglobulin (Ig) G, IgM, and IgA and also the intestinal IgM and IgA secretion. Moreover, in immunized rats, the intake of cocoa decreased specific IgG1, IgG2a, IgG2c, and IgM concentrations in serum. This immune-regulator potential was then tested in disease models in which antibodies play a pathogenic role. A cocoa-enriched diet was able to partially prevent the synthesis of autoantibodies in a model of autoimmune arthritis in rats and was also able to protect against IgE and T helper 2-related antibody synthesis in two rat models of allergy. Likewise, a cocoa-enriched diet prevented an oral sensitization process in young rats. In this review, we will focus on the influence of cocoa on the acquired branch of the immune function. Therefore, we will focus on how a cocoa diet influences lymphocyte function both in the systemic and intestinal immune system. Likewise, its potential role in preventing some antibody-induced immune diseases is also included. Although further studies must characterize the particular cocoa components responsible for such effects and nutritional studies in humans need to be carried out, cocoa has potential as a nutraceutical agent in some hypersensitivity status.

## Introduction

Antibody response is a kind of acquired immune response produced by complex interactions between several types of immune cells after the entry of an antigen into the body. In brief, when dendritic cells come into contact with an antigen in the skin or the mucosa, they become antigen-presenting cells and will be in charge of finding specific helper (Th) cells in order to trigger an acquired immune response (1). Activated specific Th cells will differentiate into effector T cells that, by means of different patterns of cytokines, will enhance the function of cells, such as B lymphocytes, macrophages, natural killer (NK) cells, cytotoxic T (Tc) lymphocytes, mast cells, or eosinophils. The activation of B cells, mainly related to Th2-immune response, will produce the formation of plasma cells that will eventually synthesize antibodies against the triggering antigen (1). In addition, inside the germinal centers of the secondary lymphoid tissues, another kind of antigen presentation occurs. Follicular dendritic cells (FDC) retain the native antigen that could prime B cells to synthesized specific antibodies (2, 3). The FDC can form immune complex-coated bodies known as iccosomes that could also affect B cell activation, maturation, and maintenance (4). Whereas antibodies will neutralize or facilitate antigen destruction, sometimes, such as in hypersensitivity and autoimmune reactions, they could have a harmful effect on the body.

Although the earliest evidence for the medical use of cocoa was found in Mesoamerican civilizations (5), nowadays, the healthy properties of cocoa and its derivatives are re-emerging. In addition to the effects of cocoa on cardiovascular health (6, 7), the nervous system (8), and cancer (9–11), cocoa also has an effect on the immune system. The immunomodulatory properties of cocoa include its potential anti-inflammatory role, demonstrated in both *in vitro* and *in vivo* studies (12–14). However, only few clinical studies with this aim have been carried out, and recently it was suggested that there is scarce evidence of the anti-inflammatory effects of cocoa consumption in humans (15). Nevertheless, researchers in this field have joined on several occasions to discuss in depth the effects of chocolate and cocoa on medicine and have demonstrated the increasing emergence of cocoa as a diet compound able to prevent some diseases, or even being a coadjuvant in some therapies (16, 17). In this review, we will focus on the influence of cocoa on the acquired branch of the immune function. Therefore, we will focus on how a cocoa diet influences lymphocyte function both in the systemic and intestinal immune system. Likewise its potential role in preventing some antibody-induced immune diseases is also included.

## Cocoa Influences Systemic Antibody Synthesis

Preclinical studies performed 10 years ago showed for the first time the *in vivo* influence of a cocoa diet on the immune system (14, 18). These studies were carried out in young rats that were fed a diet containing 10% cocoa or in rats that were orally administered with a dose equivalent to 4% cocoa in food intake for 3 weeks. Results showed that the 10% cocoa-enriched diet, but not the 4% dose, was able to decrease serum immunoglobulin (Ig) G, IgM, and IgA concentrations (18) (Table 1). A further analysis of IgG isotypes showed that 3-week-old rats fed a 10% cocoa diet for 3 weeks resulted in attenuated levels of IgG2b antibodies but increased levels of IgG2a (19) (Table 1). However, in a study in which the cocoa diet was given later, at 6 weeks of age, the 10% cocoa-enriched diet was associated with lower values of serum IgG2a but higher serum IgG2c concentrations than those present in animals fed the standard diet (20) (Table 1). Moreover, it was observed that the minimum dose to achieve such an effect was 5% cocoa in the diet (20) and, at any rat age, a 5 or 10% cocoa diet attenuated the serum levels of IgM and IgA (19, 20), the effects being clearer when animals were younger and the diet lasted longer. Therefore, these studies in rats showed that a cocoa diet influences systemic immunoglobulin production but the effect depends on the antibody isotype, the age of the animal, and the length of the cocoa intervention.

**Table 1:** Summary of the effects of cocoa diet in serum immunoglobulins and specific antibodies in healthy rats.

Strain	Initial age (weeks)	Cocoa dose	Length of the study (weeks)	Results	Reference
Wistar rats	3	4% by oral gavage	3	=IgG	(18)
				=IgM	
				=IgA	

		10% in the food	3	↓IgG	
				↓IgM	
				↓IgA	

Wistar rats	6	2% in the food	3	=IgG1, IgG2a, IgG2b, IgG2c	(20)
				=IgM	
				=IgA	

		5% in the food	3	=IgG1, IgG2b, ↓IgG2a, ↑IgG2c	
				=IgM	
				=IgA	

		10% in the food	3	=IgG1, IgG2b, IgG2c, ↓IgG2a	
				↓IgM	
				=IgA	

Wistar rats	4	10% in the food	7	=IgG1, IgG2a, IgG2c, ↓IgG2b	(19)
				↓IgM	
				↓IgA	

Wistar rats	3	4% in the food	9	=Specific IgG2a, IgG2b	(21)
				↓Specific IgG1, IgG2c	
				↓Specific IgM	

		10% in the food	9	↓Specific IgG1, IgG2a, IgG2c	
				↑Specific IgG2b	
				↓Specific IgM	

*Arrows indicate increases or decreases, equals sign means no changes*.

*Ig, immunoglobulins*.

Apart from the cocoa’s influence on basal serum immunoglobulin levels, it was interesting to shed some light on the antibody response in rats after a specific challenge, i.e., in immunized rats. In these conditions, animals were fed cocoa before and during an immunization process, and the overall synthesis of specific antibodies was also lowered (21) (Table 1). Specifically, the accurate analysis of antibodies revealed that the most attenuated isotypes were specific IgM, IgG1, IgG2a, and IgG2c antibodies, whereas specific IgG2b concentrations held steady or even increased with the 10% cocoa diet. As IgG rat isotypes can be associated with Th1 (IgG2b) or Th2 (IgG1 and IgG2a) immune response (22), these results may suggest a regulatory effect of cocoa in Th2-immune responses. This lowering effect on specific IgG1 and IgG2a, and therefore on Th2-related response, could be associated with cocoa polyphenols given that other polyphenols, such as genistein, chrysin, and apigenin (23, 24), and those from apple or soybean caused similar results (23, 25).

After establishing cocoa’s influence on immunoglobulin synthesis, the reason why this diet produced such an effect remained to be studied.

## Cocoa Influences Composition and Functionality of Primary and Secondary Lymphoid Tissues

To ascertain the mechanisms induced by cocoa on the antibody immune response, lymphoid tissue composition and lymphocyte functionality were then determined. In addition, as cocoa intake can interact with gut-associated lymphoid tissue (GALT), several investigations were carried out to ascertain the influence of cocoa in this particular compartment of the immune system. Preclinical studies carried out in rats demonstrated that a cocoa diet modifies lymphoid tissue composition and function (13). Lymphoid tissues are considered as primary or secondary depending on whether they are devoted either to the formation of the lymphocyte repertoire or to the development of the immune response, respectively (26). Thymus is a primary lymphoid tissue where T-cell maturation takes place, whereas lymph nodes, spleen, and mucosal lymphoid tissue belong to the secondary lymphoid tissue category (27).

### Cocoa and Systemic Lymphoid Tissue Composition

It was demonstrated that Wistar rats receiving a 10% cocoa diet for 3 weeks accumulate cocoa polyphenol metabolites in immune tissues, such as the thymus, lymph nodes, and spleen (28). In particular, the highest accumulation was in the thymus, where phenotypic changes were found due to the diet. In particular, cocoa intake resulted in an enhancement of the progression of immature thymocytes (those with low expression of the αβ T-cell receptor—TCR αβ^−^, and expressing or non-expressing the clusters of differentiation CD4 and CD8, i.e., TCRαβ^low^CD4^−^CD8^−^ or TCRαβ^low^CD4^+^CD8^+^) toward more mature stages (TCRαβ^high^CD4^+^CD8^−^) (29) (Table 2). In spite of this increase in CD4^+^ (Th) cells in the thymus, the analysis of a secondary lymphoid tissue, such as the spleen, revealed that a 10% cocoa diet in young rats for 3 weeks increased the proportion of spleen B cells and decreased that of Th lymphocytes (18) (Table 2).

**Table 2:** Summary of the effects of cocoa diet in lymphocyte composition of lymphoid tissues.

Lymphoid tissue	Cocoa dose	Length of the diet (weeks)	Results (% cells)	Reference
Thymus	10% in the food	3	↓TCRαβ^low^CD4^−^CD8^−^	(29)
			↓TCRαβ^low^CD4^+^CD8^+^	
			↑TCRαβ^high^CD4^+^CD8^−^	

Spleen	4% by oral gavage	3	No changes	(18)
	10% in the food		↑B	
			↓Th	

Lymph nodes	4% by oral gavage	3	No changes	(30)
	10% in the food		↑TCRγδ^+^	
			↓Th	
			↑Tc	

	10% in the food	4	↑NK	(31)
			↑B	
			↓TCRαβ^+^	
			↑TCRγδ^+^ (↑CD8αα^+^)	
			↓Th (↓CD62L^+^)	
			↑Tc (↑CD25^+^, ↑CD103^+^, ↓CD62L^+^)	

	10% in the food	6	↓TCRαβ^+^	(32)
			↓Th	
			↑Tc	
			=Treg	

Peyer’s patches	4% by oral gavage	3	No changes	(30)
	10% in the food		↑B	
			↓TCRαβ^+^	
			↑TCRγδ^+^	
			↓Th	

	10% in the food	4	↑TCRγδ^+^	(33)
			↑NKT	
			↓Th (↑ CD25^+^, ↑CD103^+^, ↓CD62L^+^)	
			=Tc (↑ CD103^+^)	

Intestinal intraepithelium	10% in the food	4	↑TCRγδ^+^	(33)
			↑NK	
			↓TLR4^+^	
			↑CD4^+^CD103^+^	

Intestinal lamina propria	10% in the food	4	↓NKT	(33)
			↓IgA^+^	

*Arrows indicate increases or decreases, equals sign means no changes*.

*CD, cluster of differentiation; TCRαβ^low^, cells with low expression of αβ T-cell receptor; Th, T helper lymphocytes; Tc, T cytotoxic lymphocytes; TCRγδ^+^, cells with γδ T-cell receptor; NK, natural killer cells; NKT, natural killer T cells; Treg, T regulatory cells; TLR, toll-like receptor; Ig, immunoglobulin*.

Lymph nodes were also affected by a cocoa diet. In particular, in mesenteric lymph nodes, a cocoa-enriched diet for 3 or 4 weeks in rats increased the proportion of innate cytotoxic lymphocytes, such as cells expressing γδ T-cell receptor (TCRγδ^+^) and NK cells, and also that of the Tc lymphocytes and B cells, whereas the proportion of Th cells decreased (30, 31) (Table 2). These effects were only produced by a 10% cocoa diet whereas a 4% cocoa dose was insufficient to influence the phenotype of mesenteric lymph nodes (30). Similarly, the intake of a 10% cocoa-enriched diet given to rats for 6 weeks decreased the proportion of TCRαβ^+^ cells but did not modify that of regulatory T cells (Treg) in inguinal lymph nodes in rats (32) (Table 2).

A more in-depth analysis of lymphocytes in mesenteric lymph nodes revealed that the increase of TCRγδ^+^ cells was attributed to the presence of a higher amount of CD8αα^+^ cells, a typical intestinal phenotype, which could be due to the migration of this cellular type from the intestine (34). The increase of Tc cells in mesenteric lymph nodes was accompanied by a higher proportion of activated cells (CD25^+^CD8^+^ cells) and cells expressing the αE-integrin (CD103^+^CD8^+^ cells) and a lower proportion of cells bearing L-selectin (CD62L^+^CD8^+^ cells) (31) (Table 2). CD103 is a subunit of the αE-integrin that can mediate cell adhesion and migration to the gut (35), whereas L-selectin is involved in lymphocyte rolling on the endothelium and the homing to secondary lymphoid tissues (36). These results could mean that the cocoa diet decreased the arrival of blood lymphocytes to mesenteric lymph nodes whereas it may favor intestinal cells entering. As cocoa compounds can reach the small intestine and even the colon (37, 38), they can affect the intestinal lymphocytes and promote their migration to mesenteric lymph nodes.

Overall, the increased proportion of CD8αα^+^TCRγδ^+^ cells, NK cells, and CD103^+^ Tc cells in mesenteric lymph nodes could be involved in cocoa’s influence on antibody immune response. TCRγδ^+^ cells have been associated with an attenuating effect on the synthesis of antibodies (39), and NK cells could also contribute to the regulation of antibody synthesis (40). Moreover, CD103^+^ cells have been associated with a regulatory function given that their proportion increased after treatment with immunosuppressive agents (41).

After feeding a cocoa-enriched diet, cocoa flavonoid metabolites are stored in the lymphoid tissues (thymus, lymph nodes, and spleen) as well as in the liver. In fact, epicatechin metabolites have been reported to be accumulated in concentrations twofold higher in the thymus, testes, and liver than in lymph nodes and spleen (28). With regard to the liver, the 10% cocoa intake in rats enhanced hepatic antioxidant capacity, without modifying hepatic superoxide dismutase and catalase activities (29).

### Cocoa and Lymphocyte Function

The development of the acquired immune response implies the involvement of complex interactions between immune cells by means of particular surface molecules and the secretion of cytokines. The gene or protein expression of those molecules involved in the immune synapses, as well as cytokines and other molecules secreted by immune cells, can be evaluated.

**Figure 1:**
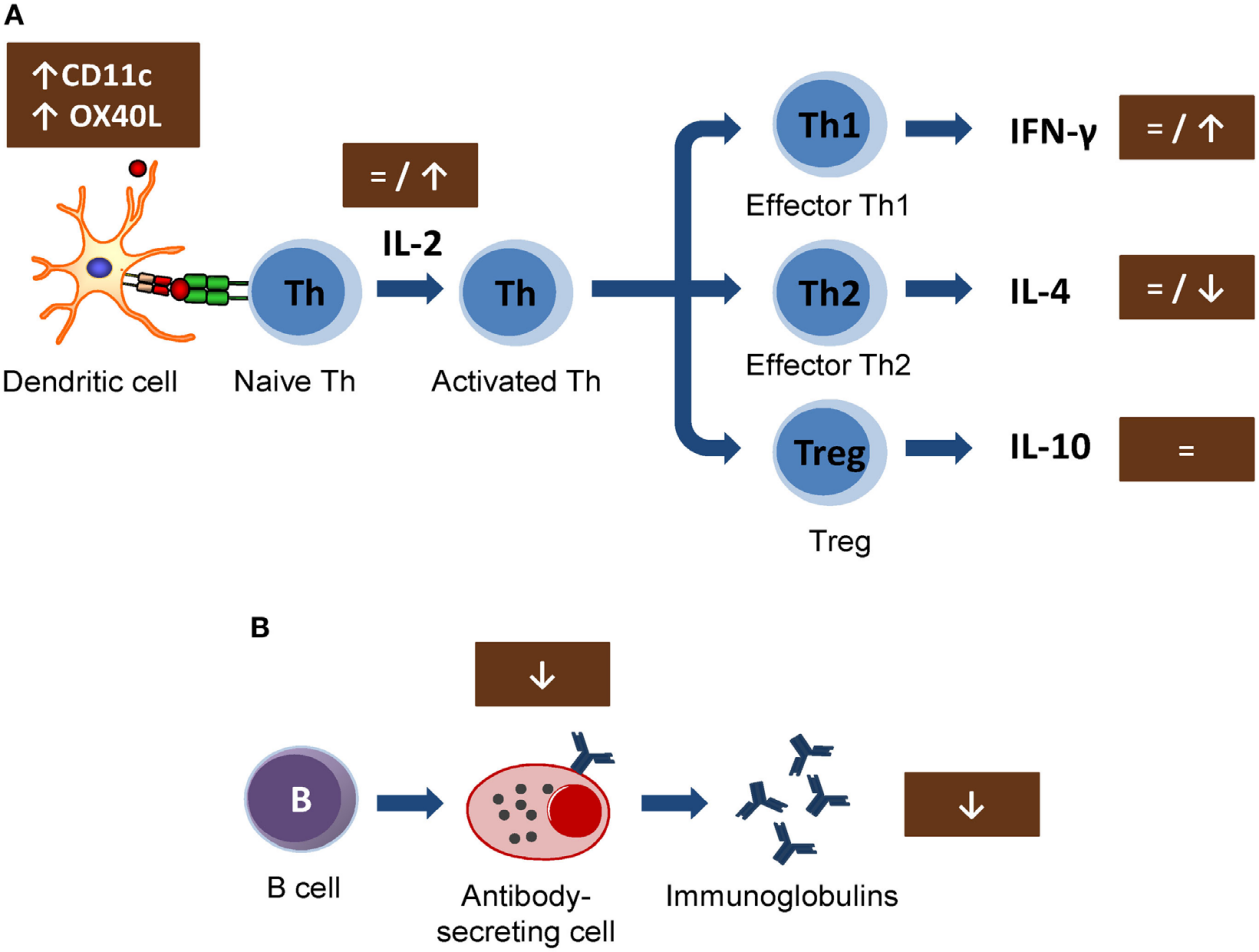
Summary of the mechanisms involved by cocoa diet on lymphocyte function: **(A)** Cocoa effect on the induction of acquired immune response, involving from antigen presentation until the development of effector T cells. **(B)** Cocoa effect on the B-cell activation and antibody production. Arrows indicate increases or decreases, equals sign means no changes. Th, T helper cells; Treg, T regulatory cells; IL, interleukin; IFN, interferon; CD, cluster of differentiation.

*In vitro* studies carried out in lymphoid cell lines showed the ability of cocoa to reduce the synthesis of interleukin 2 (IL-2) involved in early T lymphocyte proliferation (42, 43). This cytokine is mainly produced by Th cells after antigen activation (44) and plays a crucial role in immune response, enhancing Tc cell, NK cell cytotoxic activities, T cell differentiation, and stimulating the proliferation and the antibody synthesis (45). These effects could be responsible for the cocoa downregulation of the antibody synthesis. However, the results obtained *in vivo* on IL-2 secretion or lymphocyte proliferation could not confirm such a mechanism (18, 21, 30) (Figure 1A). In particular, IL-2 secretion was not modified in spleen cells from rats fed 10% cocoa for 3 weeks, even though lymphocyte proliferation increased (18). On the other hand, higher or unmodified amounts of IL-2 secretion were detected after the stimulation of lymph node cells of rats fed a 10% cocoa diet for 3 or 9 weeks (21, 30). Therefore, the interaction of a cocoa diet in the initial phases of immune activation seems not to explain the attenuating effect on antibody synthesis. However, a recent study on the gene expression of mesenteric lymph node cells shows that certain molecules present on antigen-presenting cells (dendritic cells) were modified by this diet. In particular, a cocoa diet increased the gene expression of CD11c and OX40L (31) (Figure 1A). It has been suggested that, in a model of oral sensitization, a subset of dendritic cells (CD11c^+^, CD103^+^, and CD8^+^) that migrates and activates in the mesenteric lymph nodes seems responsible for the Th2 polarization in this model (46). OX40L–OX40 interaction has been related to follicular Th cells and promotes the generation of Th2 response during antigen presentation (47, 48), and it was increased in an oral sensitization process (31). Despite these results, the cocoa diet attenuated the antibody synthesis and, therefore, this diet must interact with downstream pathways of the Th2-immune responses that would eventually inhibit antibody synthesis.

In general, the cytokine pattern secreted by activated lymphocytes reveals the stimulation of Th1, Th2, Th17, or Treg cells (49). Interferon γ (IFN-γ) is the most representative cytokine in Th1 activation (50). No changes were detected either in serum levels of IFN-γ in rats fed cocoa for 4 weeks (32) or in the secretion of IFN-γ by activated splenocytes or lymph node cells from rats fed a cocoa diet for 3 weeks (18) or 4 weeks (31). Nevertheless, an increase of IFN-γ was observed in lymph node lymphocytes from rats fed a cocoa diet for 8 weeks (21) (Figure 1A). Therefore, it seems that a cocoa intake over longer periods promotes Th1 immune response.

More interesting results were found in IL-4, the most representative Th2 cytokine (51). A reduction of IL-4 was found in activated lymph node cells from rats fed cocoa for 3 weeks (30) and in splenocytes from rats fed cocoa for 3 or 8 weeks (18, 21). However, no changes in IL-4 were found after 4 weeks of nutritional intervention (31). On the other hand, this downregulation on IL-4 secretion did not match with previous data *in vitro* (42, 43, 52) but it partially explains the down-modulatory role of the cocoa diet on antibody synthesis (Figure 1A). IL-4 promotes IgE upregulation and increases intestinal permeability (53, 54); therefore, the decrease in IL-4, along with the TCRγδ^+^ cell increase induced by the cocoa diet, may be beneficial in reducing certain stages of hypersensitivity, such as food allergy. However, some reports focused on IL-10, a regulatory cytokine (55), did not detect any modification by the 10% cocoa diet (30, 31).

The effects of cocoa lowering IL-4 secretion in some lymphocytes populations agree with those found when the specific antibody-secreting cells after an immunization were enumerated. A significant decrease in the specific IgG-secreting cell numbers was reported by 5 and 10% cocoa diets, either in spleen or lymph node tissues, although no changes were observed in specific IgM-secreting cells (21) (Figure 1B). In summary, a cocoa-enriched diet plays an immune-regulatory role in the antibody immune response to an antigen that involves a lower number of specific antibody-secreting cells and, therefore, a decrease in antibody synthesis.

## Cocoa Intake Influences Intestinal Immune System

### Cocoa Intake and Intestinal Immunoglobulins

Several years ago, Ramiro-Puig et al. first demonstrated that a cocoa-enriched diet influences the GALT by means of the modulation of the intestinal secretory IgA (S-IgA) (30). Feeding just a 4% cocoa-enriched diet caused a decrease in the fecal S-IgA levels in the second week of the diet, but they were restored at the end of the third one. The 10% cocoa intervention caused lower fecal S-IgA secretion throughout the study (30), and this effect remained when the diet was maintained for 7 weeks (19). However, when a dose–effect study was performed with diets containing 2, 5, or 10% cocoa, the 2% diet was not enough to modify intestinal immunoglobulins (20). With regards to the gut wash—a typical sample used to evaluate intestinal immunoglobulins that consists of incubating the intestine with saline buffer at 37°C in a shaker for a few minutes to allow the release of the mucosa-linked antibodies—a lower secretion of S-IgA and S-IgM was detected (19, 20, 30, 56). These results evidence a lack of the S-IgM compensatory mechanism in certain states of S-IgA deficiency (57), probably because cocoa is also acting on S-IgM. Other studies have confirmed the previous attenuating effect of a 10% cocoa diet on S-IgA levels both in fecal samples and in gut washes (31, 58). Moreover, the immunoglobulin content has also been determined in intestinal tissues, such as Peyer’s patches (PP) and mesenteric lymph nodes; and, in both tissues, the 10% cocoa diet for 3 weeks was able to decrease the levels of IgA and IgM (56).

The downregulation of intestinal immunoglobulins produced by a cocoa diet may be due to the influence of some cocoa compounds on the complex immune response developed in the GALT. This immune compartment includes inductive sites (PP and mesenteric lymph nodes) and effector sites [lamina propria (LPL) and intraepithelial lymphocytes (IEL)] (59). As explained, cocoa intake induced some changes in mesenteric lymph nodes, but the cocoa effect is not only restricted to that particular compartment. Therefore, further studies were then focused on looking in more depth the effect of a cocoa diet on PP as well as LPL and IEL.

### Cocoa Intake and Lymphocyte Composition in Small Intestine and Colon

The attenuation of serum or intestinal immunoglobulin synthesis may be the result of multitude pathways, but the reduction of mucosal IgA observed after cocoa dietary intervention may possibly involve specific mechanisms located at the intestinal site.

The rat intake of 10% cocoa for 4 weeks modified the composition of lymphocytes in the PP and in the intraepithelial compartment whereas no modifications were seen in LPL (33) (Table 2). With regard to PP, cocoa-enriched diets were able to reduce the proportion of TCRαβ^+^ T cells and to increase the proportion of B lymphocytes and TCRγδ^+^ cells (30, 33), results that agree with changes detected in the mesenteric lymph nodes (30, 31). Analyzing in depth TCRαβ^+^ cells in the intestine, the cocoa diet decreased the proportion of Th cells and increased that of natural killer T cells (NKT). In addition, after cocoa intake, PP also had higher proportions of CD4^+^CD25^+^ cells, CD4^+^CD103^+^ cells, CD8^+^CD103^+^ cells, and CD4^+^CD62L^+^ cells. Apart from the influence of cocoa intake on PP composition, the intraepithelial compartment was also affected by this diet. In IEL from the small intestine of rats fed cocoa, there was a higher percentage of TCRγδ^+^ cells (both CD8αα^+^ and CD8αβ^+^) and NK cells (33).

In summary, in the GALT, the lower production of intestinal antibodies was accompanied by a relative increase in B cell numbers and a relative decrease in TCRαβ^+^ or Th cell numbers in the inductive sites (mesenteric lymph nodes and PP). These results suggest that the antibody synthesis in B cells might be depleted by a lower stimulation from Th cells and/or a higher regulatory effect induced by cells, such as TCRγδ^+^, NK, NKT, CD4^+^CD25^+^, CD4^+^CD103^+^, CD8^+^CD103^+^, and CD4^+^CD62L^+^, which is in agreement with the role of some of these cells in the regulation of the antibody synthesis (25, 40, 60). In whatever way the activation and differentiation of intestinal B cells was attenuated, a depletion of the high-capacity IgA-secretory cells was produced as reported when they were counted by Enzyme-Linked ImmunoSpot in PP (30) or by an immunofluorescence analysis in the small intestine lamina propria (33). These results agree with a lower IgA gene expression in PP and small intestine seen after 4 and 7 weeks of cocoa intake (19, 33).

### Effects of Cocoa Diet on T Cell-Dependent Intestinal Immune Function

The gene expression of molecules involved in the intestinal immune response can shed some light on the mechanisms induced by cocoa on the regulation of the intestinal immune system. In this context, the mRNA levels of IgA, transforming growth factor (TGF) β1, IL-6, CD40, C–C chemokine receptor (CCR) 9, retinoic acid receptor (RAR) α, and RARβ have been reported in GALT tissues, such as mesenteric lymph nodes, PP, and small intestine after 3 or 7 weeks of a cocoa diet (19, 20).

**Figure 2:**
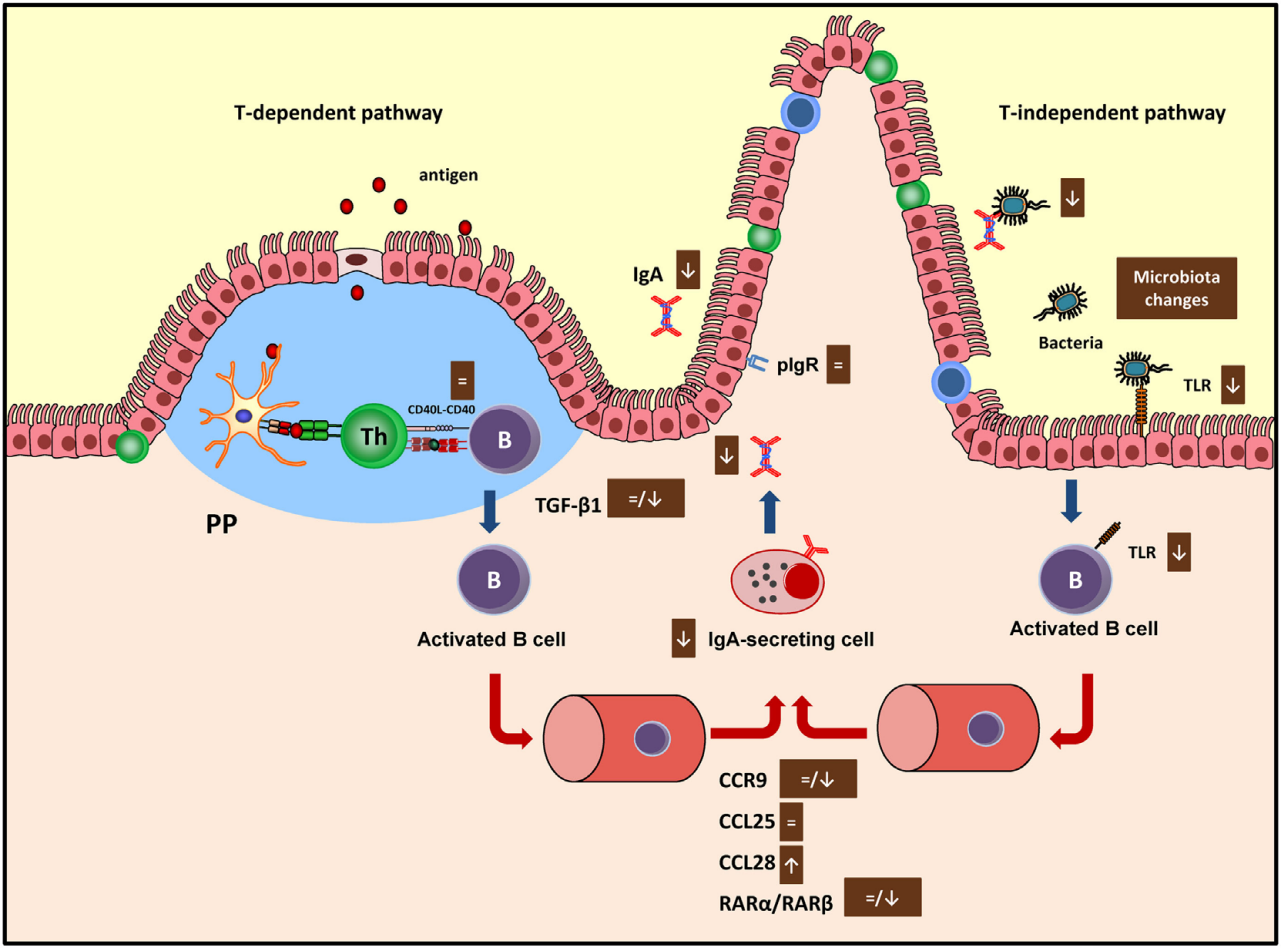
Summary of the mechanisms involved by cocoa diet on intestinal immune function in both T-dependent and T-independent pathways. Arrows indicate increases or decreases, equals sign means no changes. Ig, immunoglobulin; PP, Peyer’s patches; pIgR, polymeric immunoglobulin receptor; TLR, toll-like receptor; CCR, C–C chemokine receptor.

CD40 is involved in the interaction between B and Th cells to begin the antibody immune response (61), and cocoa intake did not modify the expression of this molecule in any of the tissues considered (19, 20) suggesting that cocoa had no influence in this phase of the antibody synthesis. The main pathway that brings differentiation of B cells into IgA-secreting cells takes place in PP or mesenteric lymph nodes (62) and depends on cytokines, such as TGF-β1 and IL-6, among others (63). The 10% cocoa diet significantly decreased the TGF-β1 expression in the small intestine after 3 and 4 weeks (20, 33), although no changes were found after 7 weeks of nutritional intervention (19) (Figure 2). On the contrary, the longest nutritional intervention, but not the shortest one, was able to downregulate the IL-6 synthesis in mesenteric lymph nodes (19, 20). Therefore, the effect on these two cytokines, TGF-β1 and IL-6, involved in the S-IgA secretion at different periods (64), might be partly responsible for the downregulatory effect of cocoa. Neither TGF-β1 nor IgA gene expressions were downregulated by the 5% cocoa diet (20), which also caused a reduction in intestinal S-IgA, indicating that additional mechanisms may be interfering in the intestinal S-IgA content.

The next stage occurs when the activated B cells leave the inductor sites (PP and mesenteric lymph nodes) and home to the effector sites (i.e., lamina propria), where the differentiation into IgA plasma cells takes place (65). Intestinal homing is regulated, among others, by chemokine-mediated interactions including the chemokine receptor CCR9, which binds to CCL25 and the CCL28 chemokines in the intestine (66). While 3 weeks of diet did not modify the expression of the CCR9 receptor nor the CCL25 but did increase the expression of CCL28 in the small intestine (20), 7 weeks of cocoa diet resulted in a downregulation of CCR9 and CCL28 gene expression in the same compartment (19) (Figure 2). In addition, retinoic acid produced by intestinal dendritic cells also plays a key role in gut homing (66) through the interaction with nuclear RAR (67). The gene expression of both RARα and RARβ was not modified in the intestinal tissue of rats fed cocoa for 3 weeks (20) but both decreased after 7 weeks (19) (Figure 2). Overall, these results could indicate that after being fed a cocoa diet over a long time there was an impairment of the arrival of IgA-secreting cells to the intestine because of the lack of gut-homing receptors observed. However, they do not explain the early decrease in S-IgA that was observed.

Finally, delivering IgA into the intestinal lumen depends on the transmembrane epithelial protein polymeric immunoglobulin receptor (pIgR) (68). This receptor was not modified by the cocoa intake (20), thus indicating that cocoa-induced S-IgA reduction did not occur as a consequence of a decreased transport across the epithelium.

### Effects of Cocoa Diet on T Cell-Independent Intestinal Immune Function

Apart from these IgA-secreting mechanisms that depend on T-cell activation, IgA^+^ B cells can be alternatively generated in a T-cell-independent manner involving toll-like receptor (TLR) signaling.

The gene and protein expression of other TLR has been modified both in the inductive sites of GALT (PP and mesenteric lymph nodes) and the effector sites (intestinal wall) after cocoa intake (19, 20, 33). In this context, among other components present in the cocoa, flavonoids have been suggested as dietary factors able to modulate TLR-mediated signaling pathways (69). TLR pathways can be modulated by flavonoids at different levels, and there are evidences of several flavonoids interfering at gene/protein expression level, in subsequent activation pathways such as the myeloid differentiation primary response 88 (MyD88), TIR-domain-containing adaptor protein inducing interferon beta (TRIF), and even downstream-associated signal transduction cascades (i.e., MAPK) (69). In this sense, alternative mechanisms in TLR regulation by cocoa flavonoids have been also suggested such as the direct modulation of their intracellular negative regulators such as the interleukin-1 receptor associated kinase (IRAK), toll interacting protein (TOLLIP), etc. To date, *in vitro* studies demonstrate the upregulation of IRAK-M by procyanidin dimer B2 (70), similarly to the effect described by other flavonoids like epigallocatechin-galate in TOLLIP expression (70). Anyway, the synergistic action of cocoa on all these TLR-activating signaling could also contribute to the attenuation of S-IgA synthesis.

Cocoa intake for 4 weeks reduced the proportion of TLR4^+^ cells in the IEL compartment (33) which agrees with a decrease of TLR4 mRNA in small intestine observed in previous studies (Figure 2). Nonetheless, higher TLR4 gene expression was found in PP (19). The TLR4 is the receptor of bacterial endotoxin lipopolysaccharide, and its signaling has implications for IgA production (71), becoming another pathway to attenuate intestinal S-IgA synthesis.

Toll-like receptors are expressed preferentially in tissues that are in constant contact with microorganisms (72, 73), and changes in the TLR expression induced by flavonoids could reflect changes in the intestinal microbiota and/or in its relation with intestinal immune cells (69, 74). Accordingly, several studies have shown that cocoa (58, 75, 76), cocoa flavonoids (58, 77), or cocoa fiber (78) induce changes in gut microbiota composition. Moreover, a lower proportion of IgA-coated bacteria have been observed after cocoa intake (79).

In summary, it could not be discarded that the influence of cocoa on GALT was partially mediated by its effect on the intestinal microbiota, which can lead to differential TLR activation and, therefore, may also influence the lowering IgA effect of cocoa.

Recently, an analysis of an untargeted ^1^H NMR spectroscopy-based metabolomic approach in 24-h urine samples have been carried out in order to correlate urine cocoa metabolites with cocoa effects on immunity and the gut microbiota (80). The results of this analysis demonstrate that cocoa intake, besides affecting microbiota composition, also alters the host and bacterial metabolism concerning energy and amino acid pathways leading to a particular metabolic signature that correlates with the S-IgA lowering effect of cocoa. Accordingly, a different pattern of intestinal and serum short-chain fatty acids, with increasing amounts of butyric acid, has been reported (78).

Finally, and in order to have a broader view of the molecules involved in the intestinal immune response modulated by cocoa, the changes in colonic gene expression by a microarray analysis after a cocoa nutritional intervention has been carried out (81). This study shows that a cocoa diet downregulated an extensive number of genes, many of them involved in the biological processes related to the immune system and inflammation. Specifically, the most downregulated gene after cocoa intake was tachykinin 4 (81), described as the promoter of B lineage cells (82), which could explain the attenuating effect of cocoa on antibody synthesis, despite the fact that the proportion of B cells did not decrease but, on the contrary, increased in some lymphoid tissues. Moreover, other genes involved in pathways related to the mast cell-mediated immunity, its activation, and its degranulation were downregulated (81), pointing out the possible role of cocoa in inducing tolerance in allergic processes as observed in some studies next reported.

### Cocoa Intake Also Influences Another Mucosal Lymphoid Tissue

The mucosal immune system is interconnected (59). Due to cocoa’s influence on the intestinal immune system, it became of interest to know whether this effect was also extended to other mucosal compartments, such as the salivary glands. The IgA and IgM content in the salivary glands (submaxillary and parotid salivary glands) was quantified after a 10% cocoa intake in rats for 3 weeks. The cocoa diet induced a decrease in the IgA and IgM content in both glands (56). This attenuating effect was associated with a drastic reduction in the IgA gene expression together with a lower expression of some molecules involved in the maturation and differentiation of B cells, such as IL-6 and TGF-β1 (56), as previously observed in the small intestinal samples (19, 20). However, in agreement with what was detected at intestinal level, no changes were detected in pIgR gene expression in the salivary glands. Therefore, in conclusion, this study shows that cocoa intake not only has an influence on the gut intestinal compartment and the systemic immunity but also on other mucosal sites in rats.

## Effect of Cocoa Diet on Antibody-Mediated Diseases

Due to the attenuating properties of cocoa on immunoglobulin levels after cocoa intake in rats, it was of interest to test its impact on diseases in which antibodies play a harmful effect. Therefore, this nutritional intervention was tested on animal models of arthritis and allergy.

### Effect of Cocoa Diet on Experimental Arthritis

Rheumatoid arthritis is a symmetric, polyarticular, systemic, and autoimmune inflammatory disease in which multiple factors, including genetic, immune, and environmental ones are involved (83). Diet components such as n-3 fatty acids, vitamins D and K, and antioxidants are protective compounds against rheumatoid arthritis (84). In this context, diets containing 5 or 10% cocoa were tested on adjuvant arthritis, a model of rheumatoid arthritis widely used for the screening of anti-inflammatory drugs (85). In this animal model, cocoa diet decreased the synthesis of antibodies against the pathology inducer (Table 3) and was also able to decrease the proportion of Th cells in both blood and regional lymphoid tissues (86). This latter effect is important because, as anti-CD4 therapy has been shown to prevent or ameliorate adjuvant arthritis (87, 88), the cocoa-induced decrease in Th cells could be beneficial to the arthritic process. Moreover, a 10% cocoa diet avoided the Th/Tc imbalance and the reduction of the proportion of NKT cells produced by the disease (86). However, the effect of cocoa on hind-paw inflammation was very poor (86), which did not agree with the protective effect of other flavonoids in a similar inflammatory model when given by oral (quercetin) or by intraperitoneal routes (quercetin, rutin, hesperidin, and morin) (89–91). Nevertheless, a cocoa extract inhibited mice ear edema (92) and acute paw edema in rat (93, 94). Moreover, cocoa flavonoids such as epicatechin, catechin, and procyanidin B2, among others, are able to attenuate the synthesis of inflammatory mediators, such as tumor necrosis factor (TNF)-α, monocyte chemoattractant protein-1, IL-6, and IL-8 (95–100).

**Table 3:** Summary of the effects of cocoa diet in specific antibodies in rat models of arthritis and allergy.

Model	Strain	Cocoa dose	Results	Reference
Adjuvant arthritis	Wistar	5% in the food	↓Specific antibodies	(86)
		10% in the food	↓Specific antibodies	

Collagen-induced arthritis	Louvain	5–10% in the food	=Specific IgG1	(32)
			↓Specific IgG2a, IgG2b, IgG2c	

Allergy induced by intraperitoneal route	Brown Norway	10% in the food	↓Specific IgG1, IgG2a	(22)
			=Specific IgG2b	
			↓Specific IgE	

Food allergy induced by intraperitoneal and oral routes	Brown Norway	10% in the food	↓ specific IgG1, IgG2a	(101)
			=Specific IgG2b	
			↓Specific IgE	

Oral sensitization	Lewis	10% in the food	↓Specific IgG1, IgG2b	(31)
			↓Specific IgM	

*Ig, immunoglobulin*.

*Arrows indicate increases or decreases, equals sign means no changes*.

The influence of a 10% cocoa diet was also analyzed in collagen-induced arthritis, another model of arthritis. This inflammatory model requires T- and B-cell responses to autologous collagen (102). B cells from animals with collagen-induced arthritis produce a strong specific immune response against triple helical epitopes of collagen type II (103). Anti-collagen autoantibodies bind to the joint cartilage, activate the complement cascade, and mediate the inflammatory attack on the joints, thus contributing to the disease development (104). Susceptible Louvain rats were fed with a 10% cocoa diet for 2 weeks before arthritis induction and during the latency period (2 weeks after induction), and thereafter with a 5% cocoa diet until the end of the study (an additional 2 weeks). In this case, the cocoa-enriched diet was able to reduce the synthesis of specific antibodies against type II collagen, differentially according to their isotype (Table 3), decrease the Th lymphocyte proportion in regional lymph nodes, and reduce the release of inflammatory mediators from peritoneal macrophages. However, these immunomodulatory effects were not enough to reduce the hind-paw swelling in arthritic animals (32). It must be taken into account that the decrease in anti-collagen antibody concentration in that rat strain was only observed at the end of the study, and it was in a lesser extent and more slowly than that expected and observed in healthy rats as shown before. In a similar context, other authors reported the beneficial effect of isolated flavonoids in improving the paw swelling in animals in long-term studies (105–107). Otherwise a nutritional intervention with the flavonoid genistein had no success (108).

### Effect of Cocoa Diet on Hypersensitivity Animal Models

#### Cocoa on Allergy Models

The effect of the consumption of a 10% cocoa diet over 4 weeks was studied in a model of allergy induced by an intraperitoneal (i.p.) injection of ovalbumin (OVA) and toxin of *Bordetella pertussis* in alum in young Brown Norway rats (22). The cocoa diet reduced the levels of anti-OVA IgG1 and IgG2a antibodies (Table 3), i.e., immunoglobulins related to Th2-immune response in rats, as previously mentioned. In addition, cocoa consumption decreased the serum concentrations of total and specific IgE (Table 3), which is the main immunoglobulin involved in allergic reactions. These results agree with studies performed in animal models of allergy treated with polyphenols, such as baicalein (109), quercetin (110), silibinin (111), sesamin (112), or an extract of *Kalanchoe pinnata* (*Crassulaceae*) containing several flavonoids such as quercetin (113).

To analyze the mechanisms involved in such action, cytokine secretion was quantified in mesenteric lymph nodes. Contrary to what was expected, cocoa diet increased the release of IL-4, a Th2 cytokine, and decreased that of IL-10, a cytokine related to immune-regulatory responses (22). In addition, cocoa intake induced a lower secretion of TNF-α, which has been described as a contributor to the development of Th2-mediated allergic inflammation by means of promoting the homing of Th2 cells to the site of allergic inflammation. These effects of IL-10 and TNF-α agree with those reported by other flavonoids in allergic conditions (113–115).

The influence of cocoa on the GALT makes it particularly interesting to test the effect of this nutritional intervention on a food allergy process. A model of food allergy using OVA as allergen was carried out in Brown Norway rats, combining an i.p. and oral administration of the allergen. The quantification of serum anti-OVA IgG1, IgG2a, and IgE antibodies revealed that the synthesis of these antibodies was completely prevented by the cocoa diet (101) (Table 3). In this study, a product that was richer in cocoa flavonoids was included, but it was not able to totally reproduce the same effects as the conventional cocoa-enriched diet. Therefore, it seems that cocoa flavonoids are only partially responsible for cocoa’s anti-allergy properties.

In addition, after anaphylactic shock, the increase of the serum mast cell protease II was partially prevented in the allergic group fed a cocoa diet (101). Nevertheless, other markers of anaphylaxis were not modified by the cocoa intake (body temperature and motor activity), suggesting that its modifications were not enough to prevent the food allergy reaction induced (101).

In order to shed light on cocoa’s anti-allergy properties, the expression of some small intestinal genes were quantified (101). The food allergy induction increased the IgA gene expression, an effect that was prevented by a cocoa diet. Moreover, the allergic animals fed a cocoa diet also had lower mRNA levels of high-affinity IgE receptors (FcεRI), mast cell protease-II, and TGF-β1 than reference animals, molecules which could be involved in the protective effect of cocoa on food allergy. Accordingly, the inhibitory effects of flavonoids on the FcεRI surface molecule or gene expression *in vitro* were described (116, 117), and the genetic analysis of colon from rats fed cocoa assessed by microarray analysis showed the downregulation of genes involved in pathways related to mast cell activation and degranulation (81). The cytokine production of food-allergic animals was also determined in mesenteric lymph nodes and spleen (101). In these tissues, the food allergy induction increased the secretion of Th2-cytokines, such as IL-4, IL-5, and IL-13. However, the cocoa diet prevented an increase in IL-5 and IL-13 in lymph node cells and that of IL-4 and IL-13 in splenocytes.

In conclusion, in models of Th-2 immune response stimulation, the intake of cocoa prevents the secretion of typical Th2-cytokines, the synthesis of IgE involved in mast cell degranulation, and also downregulates the IgE receptors in mast cells and intestinal mast cell activation, which are the cells responsible for the most allergy symptoms. However, such effects were not able to totally prevent anaphylactic shock.

#### Cocoa on an Oral Sensitization Model

Although cocoa intake prevented the allergic sensitization in a model of food allergy induced by i.p. and oral allergen administration (101), it remained to find out what happened when the sensitization with the allergen was produced using only the oral route. Therefore, a 10% cocoa-enriched diet was given to 3-week-old Lewis rats submitted to an oral sensitization model induced by the oral administration of OVA together with the cholera toxin (CT) as adjuvant (118). The oral administration of OVA/CT, three times per week and for 3 weeks, was able to break down oral tolerance and induce the synthesis of specific antibodies after 4 weeks from the beginning of the sensitization protocol. Although this model did not induce detectable specific IgE synthesis, Th2-immune response related antibodies were produced (118) (Table 3). Feeding 10% cocoa from the beginning of the study and throughout 4 weeks attenuated the development of specific antibodies in sensitized rats fed the cocoa diet (31). In particular, the 10% cocoa diet prevented the production of anti-OVA IgG1, IgG2b, and IgM in agreement with the effect of cocoa in a food allergy model in Brown Norway rats (101).

In addition, although the IgA concentrations were not increased in this rat oral sensitization model, in contrast to other models using the same adjuvant (63, 119), the cocoa diet decreased the total IgA in both serum and intestinal compartments. As stated in previous sections, a cocoa diet influences the proliferation, differentiation, and gut homing of IgA^+^ B cells (19, 31, 33), thus inducing a lower presence of these cells in the intestinal lamina propia (33) and, consequently, reducing the intestinal IgA development in line with what was reported in many studies (20, 79, 101). Additionally, the changes produced by the cocoa diet in both inductive and effector lymphoid tissues (see Cocoa Intake and Lymphocyte Composition in Small Intestine and Colon) might be responsible for the prevention of the oral sensitization. It is worth noting that the cocoa diet increased the proportion of TCRγδ^+^ and NK cells in three intestinal compartments (mesenteric lymph nodes, PP, and IEL), suggesting their role in the tolerogenic process. In line with this, unripe apple polyphenols induced an increase in the proportion of TCRγδ^+^ IEL in association with the inhibition of the development of an oral sensitization model (25), and it was also reported that the reduction of TCRγδ^+^ cells by the anti-TCRγβ antibody favors an oral sensitization in mice (120). Furthermore, NK cells could have regulatory functions contributing to the avoidance of sensitization in line with the reported prevention of allergic disease (121, 122).

Other changes induced by a cocoa diet could contribute to its tolerogenic effect (31, 33). Such changes include a reduced proportion of Th cells in mesenteric lymph nodes, PP in IEL, an increase in the percentage of CD103^+^ cells, a reduction of CD62L^+^ cells, and an increase in the percentage of CD25^+^ cells in PP. Cocoa intake also modulated the gene expression of several molecules both in mesenteric lymph nodes and in the small intestine (31, 33). In particular, cocoa consumption was associated with an increase in the gene expression of CD11c—a dendritic cell marker (123)—in mesenteric lymph nodes, whereas the mRNA levels of CD11c and CD11b were reduced in small intestinal samples; cocoa also upregulated the expression of OX40L in mesenteric lymph nodes (31)—mainly expressed on antigen-presenting cells (124). In this sense, the interaction of OX40–OX40L regulates cytokine production from T cells, antigen-presenting cells, NK cells, NKT cells, and cytokine receptor signaling (125). Additionally, cocoa decreased the gene expression of IL-1β—a potent pro-inflammatory cytokine (126)—in mesenteric lymph nodes, although no modifications were seen in the production of Th1 (IFN-γ and TNF-α), Th2 (IL-4), or Treg (IL-10) cytokines.

Overall, a cocoa intake, by means of its influence on the intestinal immune system, is able to avoid the sensitization to oral allergens, thus contributing to the downregulation of this hypersensitivity reaction.

#### Cocoa on an Atopic Dermatitis Model

Recently the role of a cocoa extract on atopic dermatitis has been published (127). The cocoa extract decreased the IgE levels induced by a *Dermatophagoides farinae* extract together with a reduction of atopic dermatitis symptoms. Particularly, the cocoa decreased the severity of the skin lesions, the loss of skin hydration and suppressed the infiltration of eosinophils and mast cells into the skin lesions. Moreover, an extract containing 0.25% cocoa downregulated IL-4 mRNA levels on the skin tissues, whereas an extract containing 1% cocoa decreased IL-5 gene expression at this level.

## Conclusion

In this review, we summarize the effect of a cocoa diet on the immune system of rats, particularly in the antibody response, both in systemic and mucosal (intestinal and extraintestinal) compartments. The analyses of cells involved in such responses, as well as molecules, such as cytokines and receptors, demonstrate that the effects of a cocoa diet are exerted at multiple sites: in the antigenic presentation, in the cytokines produced by effector Th cells, and in the intestinal homing of activated cells. Eventually, these actions will reduce the synthesis of most antibody isotypes, in particular Th2-associated antibodies as IgE. The relative decrease of Th lymphocytes associated with an increase in TCRγδ^+^ cells and NK cells detected in most lymphoid tissues studied suggest the involvement of these cells in the regulatory role of cocoa. The immunomodulatory potential of cocoa can be very beneficial in those diseases that involve hypersensitivity, such as allergy and autoimmune diseases. Nevertheless, although no signs of immunodeficiency were observed in the described studies, it must be considered that the attenuation of antibodies can be harmful when antibodies are needed to counteract a pathogenic antigen, such as infections, and to induce antibody-dependent cytotoxicity, phagocytosis, and complement activation. Although further research must characterize the particular cocoa components responsible for such effects, and nutritional studies in humans need to be carried out, cocoa has potential as a nutraceutical agent in some hypersensitivity status.

## Author Contributions

MC-B, MM-C, MA-G, and AF were responsible for the manuscript preparation. MM-C and MA-G contributed to the manuscript draft. MC-B mainly wrote the manuscript. FP-C and MC contributed to its critical revision.

## Conflict of Interest Statement

The authors declare that this study was conducted in the absence of any commercial or financial relationships that could be construed as a potential conflict of interest.
